# Poly[[tetra­aquabis­(μ-hydroxy­acetato-κ^4^ *O* ^1^,*O* ^2^:*O* ^1^,*O* ^1′^)-μ_2_-sulfato-κ^2^ *O*:*O*′-dicadmium(II)] monohydrate]

**DOI:** 10.1107/S1600536809017255

**Published:** 2009-05-14

**Authors:** Urszula Rychlewska, Beata Warzajtis, Mirjana Dj. Dimitrijević, Nenad S. Draskovic, Miloš I. Djuran

**Affiliations:** a Department of Chemistry, Adam Mickiewicz University, Grunwaldzka 6, 60-780 Poznań, Poland; b Department of Chemistry, Faculty of Science, University of Kragujevac, R. Domanovića 12, PO Box 60, 34000 Kragujevac, Serbia

## Abstract

The title compound, {[Cd_2_(C_2_H_3_O_3_)_2_(SO_4_)(H_2_O)_4_]·H_2_O}_*n*_, was obtained unintentionally in a transmetallation reaction. The crystal structure contains a two-dimensional metal–organic framework based on Cd^II^–(μ-hydroxy­acetato-κ^4^ *O* ^1^,*O* ^2^:*O* ^1^,*O* ^1′^)–Cd^II^ zigzag chains joined together by bridging SO_4_ anions. The resulting layers are shifted with respect to each other and are stacked along the *c* axis. Their construction is supported by hydrogen bonds between water molecules and between water molecules and carboxylate or sulfate groups. Neighbouring layers are bridged by hydrogen bonds between the hydroxyl substituent and a sulfate anion. The sulfate anion and solvent water mol­ecule are located on twofold axes. The results demonstrate that care must be taken when preparing ethyl­enediamine­tetra­acetic acid-type complexes by transmetallation, in order to avoid precipitation of metal complexes with the α-hydroxy­acetate ligand.

## Related literature

For examples of the successful application of transmetallation reactions in the synthesis of metal(II) complexes with hexa­dentate 1,3-propane­diamine­tetra­acetate and 1,4-butane­diamine­tetra­acetate ligands, see: Radanović *et al.* (2003 ▶, 2004 ▶, 2007 ▶); Rychlewska *et al.* (2000 ▶, 2005 ▶, 2007 ▶).
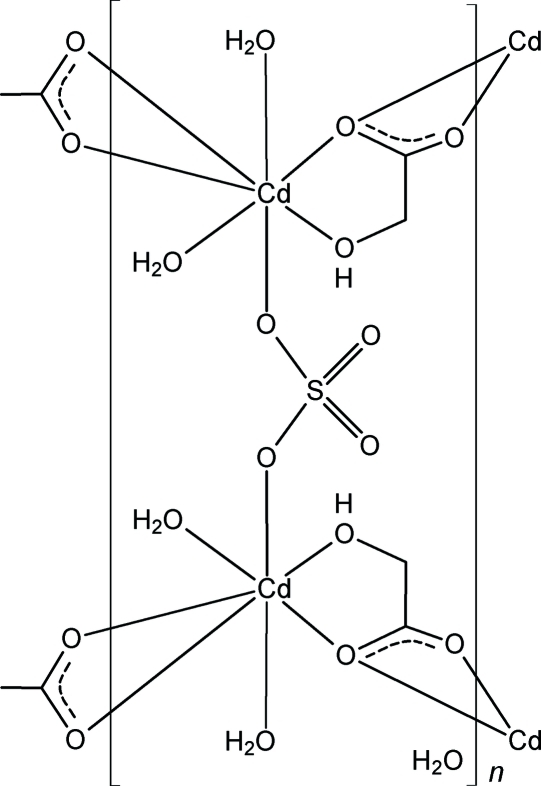


## Experimental

### 

#### Crystal data

[Cd_2_(C_2_H_3_O_3_)_2_(SO_4_)(H_2_O)_4_]·H_2_O
*M* *_r_* = 561.03
Monoclinic, 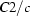

*a* = 13.5750 (3) Å
*b* = 8.5777 (1)
*c* = 13.7734 (3) Å
β = 107.528 (2)°
*V* = 1529.34 (5) Å^3^
*Z* = 4
Mo *K*α radiation
μ = 2.99 mm^−1^
*T* = 295 K
0.30 × 0.30 × 0.20 mm

#### Data collection

Kuma KM4 CCD κ-geometry diffractometer
Absorption correction: multi-scan (*CrysAlis RED*; Oxford Diffraction, 2007 ▶) *T*_min_ = 0.388, *T*_max_ = 0.550
8842 measured reflections
1710 independent reflections
1602 reflections with *I* > 2σ(*I*)
*R*_int_ = 0.018

#### Refinement

*R*[*F* ^2^ > 2σ(*F* ^2^)] = 0.015
*wR*(*F* ^2^) = 0.038
*S* = 1.13
1710 reflections
102 parameters
H-atom parameters constrained
Δρ_max_ = 0.46 e Å^−3^
Δρ_min_ = −0.31 e Å^−3^

### 

Data collection: *CrysAlis CCD* (Oxford Diffraction, 2007 ▶); cell refinement: *CrysAlis RED* (Oxford Diffraction, 2007 ▶); data reduction: *CrysAlis RED*; program(s) used to solve structure: *SHELXS86* (Sheldrick, 2008 ▶); program(s) used to refine structure: *SHELXL97* (Sheldrick, 2008 ▶); molecular graphics: *XP* (Siemens, 1989 ▶) and *Mercury* (Bruno *et al.*, 2002 ▶); software used to prepare material for publication: *SHELXL97*.

## Supplementary Material

Crystal structure: contains datablocks I, global. DOI: 10.1107/S1600536809017255/gk2209sup1.cif

Structure factors: contains datablocks I. DOI: 10.1107/S1600536809017255/gk2209Isup2.hkl

Additional supplementary materials:  crystallographic information; 3D view; checkCIF report

## Figures and Tables

**Table 1:** Selected bond lengths (Å)

Cd1—O1	2.3584 (14)
Cd1—O2	2.3013 (14)
Cd1—O4	2.2386 (16)
Cd1—O1*W*	2.2731 (15)
Cd1—O2*W*	2.3245 (14)
Cd1—O2^i^	2.5937 (14)
Cd1—O3^i^	2.3379 (17)

Symmetry code: (i) 
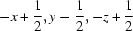
.

**Table 2:** Hydrogen-bond geometry (Å, °)

*D*—H⋯*A*	*D*—H	H⋯*A*	*D*⋯*A*	*D*—H⋯*A*
O1*W*—H1*W*⋯O2*W*^ii^	0.85	1.94	2.784 (2)	170
O1*W*—H2w⋯O3^iii^	0.85	1.88	2.723 (2)	172
O2*W*—H3*W*⋯O3*W*	0.85	1.88	2.720 (2)	172
O2*W*—H4*W*⋯O5^i^	0.85	1.82	2.648 (2)	162
O1—H1*O*⋯O5^iv^	0.85	1.81	2.659 (2)	175
O3*W*—H5*W*⋯O4^v^	0.85	2.25	2.813 (3)	124

Symmetry codes: (i) 
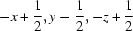
; (ii) 
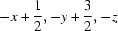
; (iii) 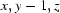; (iv) 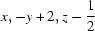; (v) 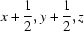.

**Fig. 1.**
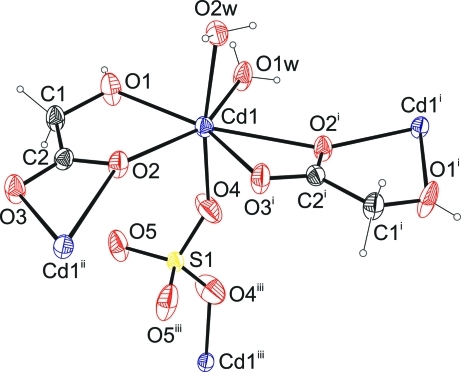
Basic supramolecular motif showing pentagonal bipyramidal coordination around CdII. Displacement ellipsoids are drawn at the 40% probability level. Symmetry codes: i = -x + 1/2, y - 1/2, -z + 1/2; ii= -x + 1/2, y + 1/2, -z + 1/2; iii= -x, y, -z + 1/2.

**Fig. 2.**
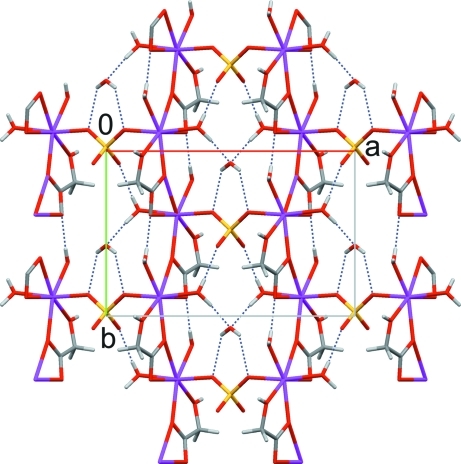
View down the monoclinic c-direction showing hydrogen bonding (dashed lines), water channels and two-dimensional structure of the complex.

## supplementary crystallographic information

### Comment

Research concerning metal–organic frameworks (MOF) has recently become important due to their potential application in a number of areas, including gas storage and catalysis. Prominent in this class of materials are frameworks that involve α-hydroxycarboxylate ligands spanning network nodes. We have recently obtained one such MOF by serendipity, when trying to synthesize cadmium(II) homometallic complex with 1,4-bdta ligand (1,4-bdta stands for 1,4-butanediaminetetraactetate) by the process of transmetallation. So far we have successfully used this strategy for obtaining *M*(II) complexes with both 1,3-pdta (1,3-propanediaminetetraacetate) (Rychlewska *et al.*, 2000; Radanović *et al.*, 2003; Radanović *et al.*, 2004; Rychlewska *et al.*, 2005, 2007) and 1,4-bdta ligands (Radanović *et al.*, 2007). In this procedure, barium(II) cations, forming the homometallic barium 1,3-pdta or 1,4-bdta complex, are being exchanged by much smaller in size metal(II) cations. At the very first step of this reaction we synthesize the ligands by reacting 1,3-propanediamine (or 1,4-butanediamine) with sodium chloracetate. It turned out that, when trying to synthesize 1,4-bdta ligand, we have obtained, in the reaction mixture, besides the expected H_4_-1,4-bdta, some amount of α-hydroxycarboxylic acid. Most probably, this α-hydroxycarboxylic acid has originated from the chloracetic acid when heated at pH > 10. Consequent addition of BaCl_2_ to the solution resulted in formation of two types of barium(II) salts, one formed by H_4_-1,4-bdta and the other by HOCH_2_COOH. An attempt to exchange barium(II) by cadmium(II) cations by addition of CdSO_4_ resulted in percipitation of two-dimensional homometallic MOF formed by Cd^II^ cations, hydroxyacetate and sulfate anions, instead of the expected Cd^II^ 1,4-bdta complex. This has been unequivocally established by the X-ray crystal structure analysis, the results of which are reported in this paper. We next attempted to verify this hypothetical synthetic route by synthesizing the very same Cd^II^ complex starting from (HOCH_2_COO)_2_Ba salt. For both samples we have performed elemental microanalyses and NMR measurements which confirmed their identity.

In the crystal the Cd^II^ nodes bind together by a singly deprotonated hydroxyacetate and doubly deprotonated sulfate linkers. The hydroxyacetate acts as a tetradentate ligand with α-hydroxyacetate group O1–C1–C2–O2 acting as a bidentate chelate to Cd1, and carboxylate group O2–C2–O3 acting as a bidentate chelate to Cd1 at *1/2 - x, 1/2 + y, 1/2 - z*. The two Cd^II^ centers sharing the same (O2) carboxylate oxygen and bridged by the O2–C2–O3 carboxylate group are 4.736 (1) Å apart, and extend in a *zigzag* manner along the *b*-direction. Each Cd^II^ cation is chelated by a hydroxyacetate residue and a carboxylate group, and is coordinated by two water molecules, and one sulfate anion, resulting in a sevenfold coordination mode (Fig. 1). The hydroxyacetate anions and one coordinated water molecule (O1W) lie in the equatorial plane while the other water molecule (O2W) and the sulfate anion take the axial positions. Hence, the coordination polyhedron formed around Cd1 is a distorted pentagonal bipyramid with the in-plane *cis* bond angles in the range 52.37 (4)–83.68 (5)°, the smallest angle reflecting chelation by the carboxylate ligand, and with the out-of-plane *trans* angle of 170.76 (6)°. Sulfate anions lie on the twofold axis and connect the neighbouring Cd^II^ centers, related by this symmetry axis *via* O4 oxygen atoms. Separation of the two Cd^II^ metal centers bridged by the sulfate group measures 6.773 (1) Å. The sulfate bridges help to extend the basic structural motif in the *a*-direction, thus forming a (001) layer (Fig. 2). This polymeric two-dimensional construction is additionally supported by hydrogen bonds (Table 2) that involve crystalline water molecule, which also occupies the twofold axis (O3w). This water molecule acts as a double hydrogen bond donor to two coordinated sulfate O atoms (O4 and its symmetry equivalent) and a double hydrogen bond acceptor from the axially coordinated water molecule (O2W and its twofold equivalent). The equatorially coordinated water molecule (O1W) acts as a hydrogen bond donor to carboxylate O3 and the axially coordinated water O2W. The neighbouring two-dimensional layers are bridged by a hydrogen bond formed between a hydroxyl group (O1) acting as a hydrogen-bond donor and the uncoordinated oxygen atom (O5) from the sulfate anion. The hydrogen bond parameters are provided in Table 2.

The reported X-ray analysis allowed us to identify and structurally characterize the unintentionally synthesized two-dimensional MOF and to demonstrate that care must be taken when preparing the edta-type complexes by transmetallation to avoid precipitation of metal complexes with α-hydroxyacetate bridges. To remove the excess of Ba(II) α-hydroxyacetate, a water washing procedure should be repeated several times.

### Experimental

2.56 g (0.003 mol) of CdSO_4_.8H_2_O were dissolved in 50 cm^3^ of distilled water at 70°C. To this solution, 6.27 g of solid Ba[Ba(1,4-bdta)].2H_2_O containing Ba(HOCH_2_COO)_2_ in ratio 3:1, respectively, was added and the reaction mixture was heated at 90°C with stirring for 6 h. The precipitated BaSO_4_ was removed by filtration and the filtrate was evaporated to *ca* 10 cm^3^ and then left in a refrigerator for several days. Colorless crystals of the title compound were collected, washed with ethanol, and air-dried. Yield 0.98 g (59.5%). The complex was recrystallized from hot water while cooling in a refrigerator. Analysis calculated for C_4_H_14_Cd_2_O_14_S.H_2_O (FW = 561.03; m. p. 416 K): C 8.84, H 2.87, S 5.90%; found: C 8.93, H 2.83, S 6.28%.

### Refinement

All H atoms attached to C atoms were placed in their idealized positions, the hydroxyl and water H atoms were located on difference Fourier maps. All H atoms were refined as riding on their carrier atoms with constraints imposed on the bond lengths and displacement parameters, *i.e.* C—H = 0.96 Å O—H = 0.85 Å with *U*_iso_(H) 20% higher than *U*_eq _of the carrier atom.

### Crystal data

**Table:**

[Cd_2_(C_2_H_3_O_3_)_2_(SO_4_)(H_2_O)_4_]·H_2_O	*F*(000) = 1088
*M**_r_* = 561.03	*D*_x_ = 2.437 Mg m^−^^3^
Monoclinic, *C*2/*c*	Melting point: 416 K
Hall symbol: -C 2yc	Mo *K*α radiation, λ = 0.71073 Å
*a* = 13.5750 (3) Å	Cell parameters from 5972 reflections
*b* = 8.5777 (1) Å	θ = 2.9–28.0°
*c* = 13.7734 (3) Å	µ = 2.99 mm^−^^1^
β = 107.528 (2)°	*T* = 295 K
*V* = 1529.34 (5) Å^3^	Prismatic, colourless
*Z* = 4	0.30 × 0.30 × 0.20 mm

### Data collection

**Table:**

Kuma KM4 CCD κ-geometry diffractometer	1710 independent reflections
Radiation source: fine-focus sealed tube	1602 reflections with *I* > 2σ(*I*)
graphite	*R*_int_ = 0.018
ω and φ scans	θ_max_ = 28.0°, θ_min_ = 2.9°
Absorption correction: multi-scan (*CrysAlis RED*; Oxford Diffraction, 2007)	*h* = −17→17
*T*_min_ = 0.388, *T*_max_ = 0.550	*k* = −10→10
8842 measured reflections	*l* = −17→17

### Refinement

**Table:**

Refinement on *F*^2^	Secondary atom site location: difference Fourier map
Least-squares matrix: full	Hydrogen site location: inferred from neighbouring sites
*R*[*F*^2^ > 2σ(*F*^2^)] = 0.015	H-atom parameters constrained
*wR*(*F*^2^) = 0.038	*w* = 1/[σ^2^(*F*_o_^2^) + (0.0212*P*)^2^ + 0.9781*P*] where *P* = (*F*_o_^2^ + 2*F*_c_^2^)/3
*S* = 1.13	(Δ/σ)_max_ = 0.001
1710 reflections	Δρ_max_ = 0.46 e Å^−^^3^
102 parameters	Δρ_min_ = −0.31 e Å^−^^3^
0 restraints	Extinction correction: *SHELXL97* (Sheldrick, 2008), Fc^*^=kFc[1+0.001xFc^2^λ^3^/sin(2θ)]^-1/4^
Primary atom site location: structure-invariant direct methods	Extinction coefficient: 0.00703 (18)

### Special details

**Table:**

Geometry. All e.s.d.'s (except the e.s.d. in the dihedral angle between two l.s. planes) are estimated using the full covariance matrix. The cell e.s.d.'s are taken into account individually in the estimation of e.s.d.'s in distances, angles and torsion angles; correlations between e.s.d.'s in cell parameters are only used when they are defined by crystal symmetry. An approximate (isotropic) treatment of cell e.s.d.'s is used for estimating e.s.d.'s involving l.s. planes.

### Fractional atomic coordinates and isotropic or equivalent isotropic displacement parameters (Å^2^)

**Table:**

	*x*	*y*	*z*	*U*_iso_*/*U*_eq_	
Cd1	0.215433 (10)	0.885016 (14)	0.174423 (9)	0.02516 (7)	
O1W	0.15455 (14)	0.67573 (17)	0.07284 (12)	0.0463 (4)	
H1W	0.1546	0.6610	0.0118	0.056*	
H2W	0.1641	0.5894	0.1046	0.056*	
O2W	0.36889 (11)	0.85773 (16)	0.13399 (12)	0.0344 (3)	
H3W	0.4134	0.9233	0.1669	0.041*	
H4W	0.3970	0.7714	0.1573	0.041*	
O1	0.14443 (14)	1.02774 (17)	0.02309 (11)	0.0499 (4)	
H1O	0.1128	1.0004	−0.0376	0.060*	
C1	0.12548 (16)	1.1879 (2)	0.03179 (14)	0.0323 (4)	
H1A	0.0528	1.2052	0.0191	0.039*	
H1B	0.1474	1.2454	−0.0178	0.039*	
C2	0.18369 (14)	1.2435 (2)	0.13702 (13)	0.0254 (4)	
O2	0.23446 (11)	1.14930 (16)	0.20257 (11)	0.0310 (3)	
O4	0.07190 (15)	0.87150 (18)	0.22208 (19)	0.0617 (6)	
S1	0.0000	0.97501 (7)	0.2500	0.02325 (13)	
O5	0.05131 (16)	1.0737 (2)	0.33543 (12)	0.0570 (5)	
O3W	0.5000	1.0723 (3)	0.2500	0.0749 (10)	
H5W	0.5380	1.1144	0.2183	0.090*	
O3	0.17860 (15)	1.38542 (15)	0.15667 (12)	0.0396 (3)	

### Atomic displacement parameters (Å^2^)

**Table:**

	*U*^11^	*U*^22^	*U*^33^	*U*^12^	*U*^13^	*U*^23^
Cd1	0.03149 (10)	0.02054 (9)	0.02099 (9)	0.00182 (4)	0.00418 (6)	−0.00095 (5)
O1W	0.0858 (12)	0.0209 (7)	0.0251 (7)	−0.0006 (7)	0.0062 (7)	−0.0006 (6)
O2W	0.0402 (8)	0.0289 (7)	0.0334 (8)	0.0051 (6)	0.0098 (6)	0.0019 (6)
O1	0.0872 (12)	0.0260 (8)	0.0193 (7)	0.0107 (7)	−0.0098 (7)	−0.0061 (6)
C1	0.0445 (11)	0.0231 (9)	0.0224 (9)	0.0022 (8)	−0.0001 (8)	0.0011 (7)
C2	0.0323 (9)	0.0211 (9)	0.0218 (8)	−0.0040 (7)	0.0067 (7)	0.0000 (7)
O2	0.0416 (7)	0.0226 (6)	0.0214 (6)	−0.0032 (5)	−0.0018 (6)	0.0001 (5)
O3	0.0629 (10)	0.0208 (7)	0.0274 (8)	−0.0011 (6)	0.0021 (7)	−0.0030 (5)
O4	0.0565 (11)	0.0353 (9)	0.1112 (18)	0.0121 (7)	0.0524 (13)	0.0067 (9)
S1	0.0263 (3)	0.0186 (3)	0.0233 (3)	0.000	0.0051 (2)	0.000
O5	0.0940 (15)	0.0310 (8)	0.0250 (8)	−0.0193 (8)	−0.0139 (8)	0.0032 (7)
O3W	0.0500 (15)	0.0271 (12)	0.140 (3)	0.000	0.0168 (17)	0.000

### Geometric parameters (Å, °)

**Table:**

Cd1—O1	2.3584 (14)	O1—H1O	0.8500
Cd1—O2	2.3013 (14)	C1—C2	1.504 (3)
Cd1—O4	2.2386 (16)	C1—H1A	0.9601
Cd1—O1W	2.2731 (15)	C1—H1B	0.9599
Cd1—O2W	2.3245 (14)	C2—O2	1.252 (2)
Cd1—O2^i^	2.5937 (14)	C2—O3	1.253 (2)
Cd1—O3^i^	2.3379 (17)	O2—Cd1^ii^	2.5937 (14)
O1W—H1W	0.8500	O4—S1	1.4543 (16)
O1W—H2W	0.8500	S1—O5	1.4468 (16)
O2W—H3W	0.8500	O3W—H5W	0.8500
O2W—H4W	0.8500	O3—Cd1^ii^	2.3379 (17)
O1—C1	1.410 (2)		
			
O4—Cd1—O1W	87.27 (7)	Cd1—O2W—H3W	110.5
O4—Cd1—O2	93.73 (5)	H4W—O2W—H3W	102.0
O1W—Cd1—O2	152.03 (5)	C1—O1—Cd1	117.86 (11)
O4—Cd1—O2W	170.76 (6)	C1—O1—H1O	107.6
O1W—Cd1—O2W	87.65 (6)	Cd1—O1—H1O	132.6
O2—Cd1—O2W	94.38 (5)	O1—C1—C2	109.54 (15)
O4—Cd1—O3^i^	92.14 (8)	O1—C1—H1A	109.8
O1W—Cd1—O3^i^	127.91 (5)	C2—C1—H1A	109.8
O2—Cd1—O3^i^	80.02 (5)	O1—C1—H1B	109.7
O2W—Cd1—O3^i^	84.91 (6)	C2—C1—H1B	109.8
O4—Cd1—O1	97.23 (7)	H1A—C1—H1B	108.2
O1W—Cd1—O1	83.68 (5)	O2—C2—O3	121.68 (18)
O2—Cd1—O1	68.45 (5)	O2—C2—C1	120.37 (17)
O2W—Cd1—O1	89.87 (6)	O3—C2—C1	117.95 (17)
O3^i^—Cd1—O1	147.55 (5)	C2—O2—Cd1	120.32 (12)
O4—Cd1—O2^i^	81.27 (6)	C2—O2—Cd1^ii^	86.96 (11)
O1W—Cd1—O2^i^	76.21 (5)	Cd1—O2—Cd1^ii^	150.69 (6)
O2—Cd1—O2^i^	131.594 (18)	S1—O4—Cd1	139.34 (10)
O2W—Cd1—O2^i^	90.02 (5)	O5—S1—O5^iii^	108.34 (13)
O3^i^—Cd1—O2^i^	52.37 (4)	O5—S1—O4^iii^	109.84 (12)
O1—Cd1—O2^i^	159.88 (5)	O5^iii^—S1—O4^iii^	112.05 (13)
Cd1—O1W—H2W	113.6	O5—S1—O4	112.05 (13)
Cd1—O1W—H1W	128.1	O5^iii^—S1—O4	109.84 (12)
H2W—O1W—H1W	109.5	O4^iii^—S1—O4	104.74 (14)
Cd1—O2W—H4W	109.2	C2—O3—Cd1^ii^	98.98 (12)
			
O4—Cd1—O1—C1	74.77 (15)	O2^i^—Cd1—O2—C2	−163.19 (14)
O1W—Cd1—O1—C1	161.19 (16)	O4—Cd1—O2—Cd1^ii^	74.85 (14)
O2—Cd1—O1—C1	−16.41 (14)	O1W—Cd1—O2—Cd1^ii^	166.05 (11)
O2W—Cd1—O1—C1	−111.16 (15)	O2W—Cd1—O2—Cd1^ii^	−100.72 (13)
O3^i^—Cd1—O1—C1	−30.9 (2)	O3^i^—Cd1—O2—Cd1^ii^	−16.67 (13)
O2^i^—Cd1—O1—C1	159.13 (13)	O1—Cd1—O2—Cd1^ii^	171.15 (15)
Cd1—O1—C1—C2	16.3 (2)	O2^i^—Cd1—O2—Cd1^ii^	−6.79 (12)
O1—C1—C2—O2	−3.4 (2)	O1W—Cd1—O4—S1	−144.9 (2)
O1—C1—C2—O3	177.33 (17)	O2—Cd1—O4—S1	7.1 (2)
O3—C2—O2—Cd1	167.52 (14)	O3^i^—Cd1—O4—S1	87.2 (2)
C1—C2—O2—Cd1	−11.7 (2)	O1—Cd1—O4—S1	−61.6 (2)
O3—C2—O2—Cd1^ii^	−1.16 (18)	O2^i^—Cd1—O4—S1	138.6 (2)
C1—C2—O2—Cd1^ii^	179.64 (15)	Cd1—O4—S1—O5	−57.4 (2)
O4—Cd1—O2—C2	−81.55 (15)	Cd1—O4—S1—O5^iii^	63.0 (2)
O1W—Cd1—O2—C2	9.6 (2)	Cd1—O4—S1—O4^iii^	−176.5 (3)
O2W—Cd1—O2—C2	102.89 (14)	O2—C2—O3—Cd1^ii^	1.3 (2)
O3^i^—Cd1—O2—C2	−173.07 (15)	C1—C2—O3—Cd1^ii^	−179.48 (13)
O1—Cd1—O2—C2	14.76 (13)		

Symmetry codes: (i) −*x*+1/2, *y*−1/2, −*z*+1/2; (ii) −*x*+1/2, *y*+1/2, −*z*+1/2; (iii) −*x*, *y*, −*z*+1/2.

### Hydrogen-bond geometry (Å, °)

**Table:**

*D*—H···*A*	*D*—H	H···*A*	*D*···*A*	*D*—H···*A*
O1W—H1W···O2W^iv^	0.85	1.94	2.784 (2)	170
O1W—H2w···O3^v^	0.85	1.88	2.723 (2)	172
O2W—H3W···O3W	0.85	1.88	2.720 (2)	172
O2W—H4W···O5^i^	0.85	1.82	2.648 (2)	162
O1—H1O···O5^vi^	0.85	1.81	2.659 (2)	175
O3W—H5W···O4^vii^	0.85	2.25	2.813 (3)	124

Symmetry codes: (iv) −*x*+1/2, −*y*+3/2, −*z*; (v) *x*, *y*−1, *z*;  (i) −*x*+1/2, *y*−1/2, −*z*+1/2; (vi) *x*, −*y*+2, *z*−1/2; (vii) *x*+1/2, *y*+1/2, *z*.
